# DNA Analysis Detects Different Mislabeling Trend by Country in European Cod Fillets

**DOI:** 10.3390/foods10071515

**Published:** 2021-06-30

**Authors:** Frederik Feldmann, Alba Ardura, Carmen Blanco-Fernandez, Eva Garcia-Vazquez

**Affiliations:** Department of Functional Biology, University of Oviedo, C/Julian Claveria s/n, 33006 Oviedo, Spain; frederik.feldmann@imbrsea.eu (F.F.); blancofmaria@uniovi.es (C.B.-F.); egv@uniovi.es (E.G.-V.)

**Keywords:** Atlantic cod, barcoding, mislabeling, European markets

## Abstract

Atlantic cod, *Gadus morhua*, is a highly appreciated fish in European seafood markets and is one of the most substituted fish species in the world. Fraud have been detected in European markets in the last decade, finding different substitute species sold as *G. morhua* or Atlantic cod on the label. In this study, we analyzed 252 samples of fresh and frozen cod fillets sold in Germany, the Netherlands, and France using DNA barcoding. Different trends were found in different countries: while the level of mislabeling found in Germany and the Netherlands remained at zero in the last years, a significant increase was found in the French markets comparing the current results with previous studies on fillets in France. On the one hand, this mislabeling proves the need to encourage European efforts to control seafood authenticity; on the other, zero mislabeling in two countries shows the success of current European regulations.

## 1. Introduction

Mislabeling of fishery products has been and still is common practice worldwide, as has been described in different studies conducted around the world in Africa [1], North America [2], South America [3], Asia [4], or Europe [5], for example. One of the reasons behind this practice is the increase of the global seafood consumption, which has doubled within the last 50 years [6]. A total of 76% per capita seafood consumption in the EU consists of wild catch products as opposed to aquaculture ones, with a subsequent price inflation for fishery products [7]. Although this trend has been drastically reversed with the COVID-19 pandemic due to the decrease of the demand for exported fishery products [8], Germany and Italy show an increase in household consumption of fresh seafood, whereas in other EU member states, the overall consumption is decreasing [9].

To allow traceability and guarantee strict product labelling for consumers within the EU, European Regulation EU 1379/2013, Art. 35, established on 11 December 2013, dictates that EU member states must publish a list of commercially traded seafood species, including scientific names. This also includes the area where the product was caught and the fishing methods used, as some methods, such as bottom trawling, can be very disruptive to the ecosystem [10]. Despite those implementations, mislabeling in the EU is still commonly reported [11]. Differences in mislabeling have been observed between different selling points, such as restaurants or catering services and retailers and supermarkets, with recent high rates of mislabeling in German mass catering [11,12]. Beyond the environmental and conservation implications, due to over- or under-representation on the catching statistics [13], mislabeling can also influence public health, as surrogate species can induce allergic outbreaks in sensitive consumers [14]. Therefore, appropriate labelling is very important from any point of view.

Identifying the species based on morphological features is often difficult for the consumer because processed fish usually lack the parts that enable their better identification, such as heads, tails, or fins, and the appearance and taste of different species might be similar. This is the reason why molecular methods, such as barcoding, are used for identification purposes. Barcoding has been proven to be effective and has also become cheaper over the last decade [15]. This process usually involves the extraction, amplification, and sequencing of different fragments, such as the mitochondrial cytochrome c oxidase I subunit gene (COI), since COI is suitable for unambiguous identification of many vertebrates, including fish at species level [16,17]; for these reasons, it is the barcode of choice employed in the first marine barcoding projects [18,19]. The DNA sequences are then edited and compared with the sequences found in data bases, such as the National Center for Biotechnology Information (NCBI) or Barcode of Life Data System (BOLD) [20].

Cod, *Gadus morhua*, is one of the most substituted species in the world [21]. Especially in the EU, the consumption of cod shows an increasing trend over the last decade, while the consumption of other species of groundfish remains generally low. It is only in the last two years that cod consumption has been declining, driven by a decline in imports and catches [8]. One factor for the decrease in catches could be the emerging cod farming industry, which, in the case of Norway, competes strongly with the fishing industry, especially since the diet of the cod is not entirely based on fish and may include soybeans [22]. Cod aquaculture has also shown to be more profitable compared to fishing, contributing to curb the dependence on the fishing industry [23]. In addition, the growing awareness of the environmental effects of large-scale fishing could contribute to cut down the consumption of fishing products. In any case, the price of Atlantic cod increased significantly in early 2020 due to Commission Regulation (EU) 2020/183, Art. 1–3, establishing a fishery closure for cod in areas 1 and 2b. This regulation might encourage incorrect labelling by introducing cheaper species to substitute the more expensive cod. Therefore, this study focuses on *G. morhua* as a target species. Nonetheless, in some countries like Belgium, recent mislabeling seems to be very low [24]. In other places like the Netherlands, no mislabeling of *G. morhua/Gadus macrocephalus* has been detected so far although this country has only been assessed once, showing the gaps of sampling efforts throughout Europe [25]. To this irregular sampling is added the fact that publications on seafood mislabeling featuring cod are scarce and inconsistent through the years. Germany, for example, has recently shown high mislabeling rates within common sole samples [26] and general mislabeling in other fish species [11,27], but not many studies have been conducted on mislabeling of *G. morhua*. Previous studies showed high percentages of mislabeling in fresh and frozen fillets/products [28,29]. In France, in 2015, the first study was developed to evaluate the extent of the mismatch between the market names and the actual species for some of the most common commercial marine fish species, including cod as one of the most often-substituted species. [30].

It is relevant to highlight the importance of knowing the mislabeling rates and the most frequent substitute species because seafood fraud not only affects the ability of consumers to make informed and sustainable seafood purchases, but it also harms fisheries and fishermen, allowing the introduction of illegal catches, or not declared ones, into the food markets [25]. There are several strategies to improve food quality control and traceability at all stages in the chain production of different product types (e.g., fresh and frozen products or recognizable or processed products) sold in different retail outlet types (e.g., supermarkets, restaurants, or fish markets). Among them, the genetic techniques used for species identification, such as the DNA barcoding, could improve the monitoring activities if used as a routine monitoring tool. However, despite its importance, there is a considerable lack of knowledge about mislabeling in different types of cod products. While recent studies reported higher occurrences of mislabeling in salted products [31], other ones do not mention the product types in detail [25]. Many studies do not specify the rate of mislabeling between the different locations when sampled in different retail outlets [5,32]. Other studies showed opposite trends: more mislabeling in supermarkets [28] or more mislabeling in fish markets [30].

Therefore, since fresh and frozen products have been found to contain mislabeled cod, the principal aims of this study are: (i) to analyze a large sample of cod products in fish markets and supermarkets of three European countries: Germany, the Netherlands and France; (ii) to compare the obtained results between countries; (iii) to compare these results with the mislabeling levels found in Europe in the last decade; and (iv) to highlight the species most commonly used as a substitute for cod.

## 2. Materials and Methods

### 2.1. Sampling and Labelling

A total of 252 fresh or frozen fillets labelled as *G. morhua* were sampled randomly from supermarkets or fish markets in Fulda, Hamburg, and Bremerhaven in Germany; Rotterdam in the Netherlands; and Sète and Montpellier in France. A total of 101 samples were taken from Germany, of which 28 were from fish markets and 73 from supermarkets. In all, 71 samples were obtained from the Netherlands, of which 30 samples were from fish markets and 41 from supermarkets. Then, 80 samples were taken from France, with 57 samples from supermarkets and 23 from fish markets. The names of the stores of purchase and the information displayed on the label, such as distributors and the catching methods, were noted (Table 1). For all samples, the scientific name was displayed on the label. Since some samples were sold as *G. morhua* and *G. macrocephalus* as one product, it was decided to include *G. macrocephalus* in the study as well. Tissue samples were cut from the fillets and stored in 5 mL Eppendorf tubes with twist-off caps filled with 98% ethanol to avoid spoilage during the travel.

### 2.2. DNA Extraction

DNA extraction was done following the chelex protocol described by Estoup et al. [33]. Briefly, a small sample of the tissue was cut and placed in a 1.5 mL tube; then, 500 μL of chelex and 7.5 μL of proteinase k were added. The samples were then placed in an oven at 55 °C for 1 h ½ and shaken every 15 min. To inactivate the proteinase k, the samples were heated at 100 °C for 20 min. The samples that did not give a successful DNA yield were re-extracted using the DNAeasy tissue kit (Qiagen, Hileden Germany) following the manufacturers’ protocol.

### 2.3. PCR and DNA Sequencing

A DNA region within the Cytochrome Oxidase subunit I gene (COI thereafter) was amplified using the primers FISHCO1 described by Handy et al. [34] and successfully applied in cod [29]:

FISHCO1LBC: 5′-TCAACYAAT CAYAAAGATATYGGCAC-3′
FISHCO1HBC: 5′-ACTTCYGGGTGRCCR AARAATCA-3′

This primer set was used to streamline the sequencing but also to allow for a longer read in the COI gene [34]. The PCR was performed following the protocol described by Pinto et al. [29].

In order to confirm the genetic identification, a second marker, the universal primers described by Ward et al. [35], were used to amplify a region within the gene coding for the COI. The PCR was adjusted and performed in a final mixture of 20 µL and included 1 µL of each primer (10 µM), 4 µL of Taq buffer, 2 µL dNTP (2.5 mM), 2 µL MgCl_2_ (25 mM), 0.15 µL Taq polymerase (5 U/mL), and 7.85 µL water. The thermal profile for all primers was as follows: the first step at denaturing 94 °C for 5 min, 35 cycles of 45 s at 94 °C, 45 s at 50 °C, and 72 °C at 1 min. The final elongation step was set at 72 °C for 4 min.

For visualization of the amplified PCR products, an electrophoresis (2% agarose and 2.5 µL SimplySafe, EURx, Poland) was performed using 4 µL of ladder and 4 µL of DNA.

The samples were sent to Macrogen Inc. (Madrid, Spain) to sequence the amplified PCR products using the Sanger sequencing method procedure.

### 2.4. Data Analysis

In order to generate consensus sequences for each specimen, the obtained chromatograms from the sequencing were edited using BioEdit v7.2.6.1. [36], and the quality of the peaks was visually checked. The presence of NUMTs was checked with MEGA-X software and GenBank database, checking the coding frame before to make the accession numbers available for the scientific community. The edited sequences were uploaded to the BLASTn search tool in GenBank (www.ncbi.nlm.nih.gov, National Institutes of Health de Estados Unidos, 15 January 2021) to identify the species of the sequence obtained. The species of the best-match hit was chosen as putative species, using an identity threshold >97% for COI barcode, as indicated in Ward et al. [35]. Sequences labelled as voucher specimen were used since this ensures a physical copy of the specimen. In the case that no voucher specimen was found in the NCBI database, sequences with reference title of a published article were used.

The information declared on the label and/or by the sellers, such as the common and scientific name of the species of the fish product sold on the markets (Table 1), was compared to the results derived from the molecular identification analysis. A few samples with best-match bacteria sequences were labelled as degraded, meaning that the DNA of the fish sample was too disintegrated by the bacteria, and discarded.

Haplotype analysis was conducted using DNAsp in order to upload one sequence per haplotype obtained in this study of *G. morhua* or *G. macrocephalus* in NCBI.

The conservation status and annual fishery quotes of the substitute species found in literature were derived from the IUCN red list and the FAO annual catching report.

### 2.5. Statistical Analysis

A linear regression analysis of the variable “% of mislabeled samples” over time since the first mislabeling report in 2008 was conducted to assess spatial trends. A chi-square analysis was performed to check changes of mislabeling in cod fillets between current (our study) and past samples in the countries considered. For both statistical tests, the statistical significance was set to 0.05. We included data from literature and the data derived from our samples.

Statistical analysis was conducted in Microsoft EXCEL. To visualize the data the statistical R (version 3.6.1) software, QGIS and Microsoft EXCEL were used.

## 3. Results

### 3.1. DNA Analysis

Of the 252 samples collected for this study (Table 1), 203 samples were successfully extracted and amplified, and negative controls were performed. From the successfully extracted samples, 171 (84.2%) of the obtained sequences from this analysis compared to NCBI gave successful matches ranging between 97–100% of pairwise sequences identity with reference sequences present in the database. Five samples showed best hits with *G. morhua* sequences but with identity below 97%, and 18 samples were below the 90% pairwise sequence identity. Since they did not display unambiguous chromatograms (some nucleotide peaks were not clear), they were labelled as degraded. The negative controls run along the PCRs were all negative, demonstrating lack of contamination during the laboratory process.

All the samples from Germany and the Netherlands analysed in this study, for which barcodes were obtained, were correctly labelled regarding the species declared on the label. Nine of 26 samples (25%) from France were mislabeled, displaying haplotypes from *Melanogrammus aeglefinus.* Haplotypes obtained for *G. morhua* matching 100% to previous uploaded haplotypes are available on the GenBank database (www.ncbi.nlm.nih.gov, 15 January 2021): *G. morhua* GB-AN (MW020365–MW020375).

### 3.2. Catch Location and Catch Method

All samples that displayed catch areas on the labels had them within FAO 27 as catch location for *G. morhua* and FAO 67 for the catch location of *G. macrocephalus* (Table 1). The most common catch method reported was trawl nets. None of the fish obtained in Germany was caught from the North Sea, whereas the fish purchased in the Netherlands had North Sea as the catch location labelled on the product. All the samples from France were labelled as *G. morhua*, contained information about the catch method, and the capture area was stated as FAO 27 in some samples and North, Barents, and Norwegian seas in others (Table 1).

#### 3.2.1. Proportion of Mislabeling over Time

Observing the evolution of cod mislabeling (*G. morhua/G. macrocephalus*) within the EU over the last decade, including our samples in 2020 (Table 2), the linear regression was negative and statistically significant (y = −0.021x + 41.543, r^2^ = 0.44; 9 d.f.; *p* = 0.04).

On the other hand, while the mislabeling in cod fillets was 0 in Germany and the Netherlands in the last decade, and it was still 0 in 2020, the changes in French markets was statically significant (*χ*^2^ = 17.79; df = 1; *p* < 0.001), with 2 cases of mislabeling found over 95 samples analysed taken from the bibliography (Table 2) and 9 cases of mislabeling found over 36 samples barcoded in this study.

#### 3.2.2. Substitute Species

**Figure 1:**
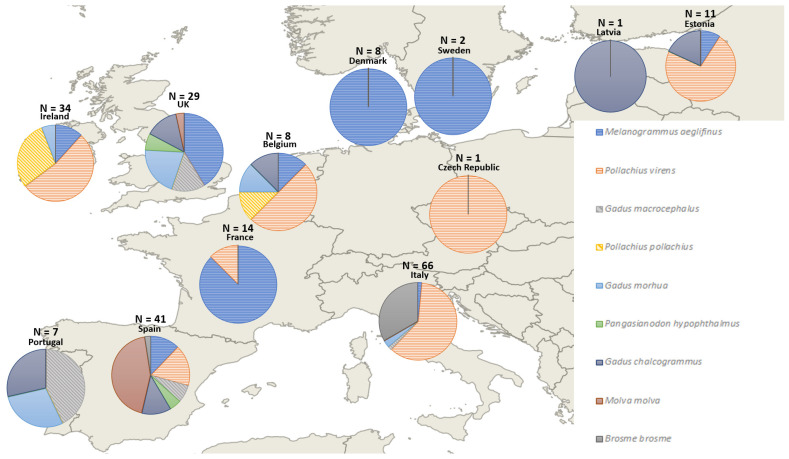
Proportion of substitute species identified in the different countries from cod samples analyzed through DNA barcoding since 2008. Only substitute species occurring in at least 3 different publications or having at least N = 10 were used in this figure.

The analysis including previous and current results showed differences in the substitute species among countries, considering only recurrent substitutions (substitute species appearing only once, in one study, were not taken into account in this analysis for simplicity). Especially, saithe (*Pollachius virens*) has been often found as a substitution species, making up the majority of substitute species in Italy, Ireland, Belgium, and Estonia. Haddock (*M. aeglefinus*) has been determined to be the dominant substitute species in France, Sweden, and Denmark, being the only substitute species in Sweden and Denmark and 84.6% of the substitute species found in France. The United Kingdom has high proportions of *G. morhua, G. macrocephalus*, and *M. aeglefinus* as substitute species. Both ways, *G. morhua* and *G. macrocephalus* were substituted for each other. Relatively high percentages of ling (*Molva molva*) were found in Spain, whereas *Pollachius pollachius* was found to be used more frequently in Ireland. The number and variety of substitute species was not the same in all countries, the highest diversity occurring in Spain, followed by Belgium and the United Kingdom (Figure 1). The different substitute species found on different years are summarized on the Table 3. *P. virens* and *M. aeglefinus* were found in nearly all papers through the years, including this current study, followed by *Gadus chalcogrammus.*

## 4. Discussion

Mislabeling is a challenge to biological diversity. Since seafood products are becoming increasingly diverse in a globalized world, it is harder for the consumer to accurately identify the products, which makes it more crucial to correctly label them [21]. This also applies to processed seafood or even fillets, as sampled for this study. Zero mislabeling in unrecognizable cod products (fresh and frozen) was found in samples from 15 selling points in different German cities and 7 in Rotterdam. However, nine (25%) of our samples collected from eight different selling points in two cities in France were mislabeled. Although a higher mislabeling in Germany due to recent peaks of species substitution in seafood [11,26,27], most of them in restaurants, could be expected, it was not found in our study for fillets. In the same way, the expectation on finding mislabeled Atlantic cod in the Netherlands due to the reduced fishing quotas as stated in Commission Regulation (EU) 2020/123, Art. 1–3 and increased value of the fish also was not confirmed. Only in France was mislabeling found. It had been reported in fillets in this country with a modest 2% of mislabeling [5,25], but it increased significantly, up to 25%, in the current study.

**Figure 2:**
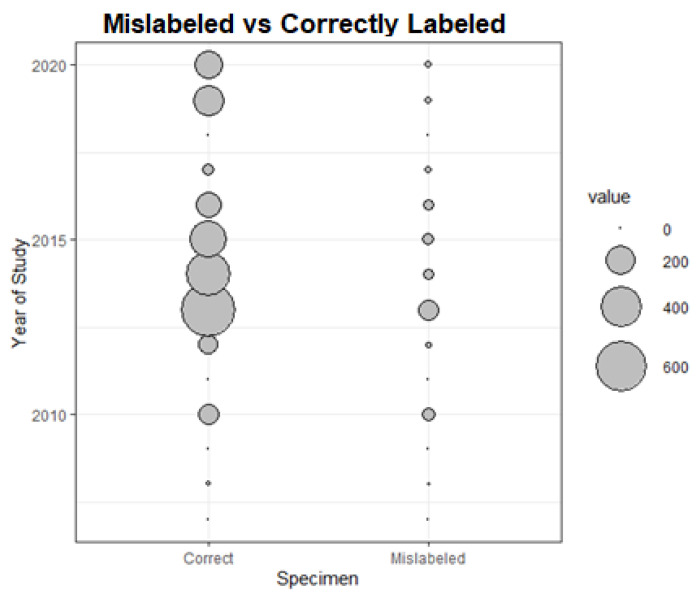
Mislabeled cod products in comparison to correctly labelled products over time within EU countries; value = sample size.

On the other hand, the outcome of this study would partially confirm previous studies, such as that of Mariani et al. [5], who, based on the rather low rate of mislabeling around 2014, suggested that seafood mislabeling within the EU was generally decreasing, as our Figure 2 shows for cod. Our results suggest that this would not be entirely true for all the countries in 2020. This was also proposed by Brechon et al. [25], who found low levels of mislabeling in cod products but also found a high variation between sampled countries, with Denmark and Estonia displaying higher mislabeling rates than the other EU countries. However, no previous studies on cod mislabeling have been conducted in both countries; thus, the country trend could not be known. This can be explained in country-specific dynamics on a governmental level [25] but also from the different levels in the awareness of substitution fraud in different countries driven by media exposure [46]. Countries such as Ireland, in which high mislabeling rates have been exposed [31] in the first wave of mislabel reports between 2008 and 2010 (there are no references for years prior to 2008), displayed lower mislabeling levels in supermarkets and fish markets later [5]. However, this is not necessarily the case for restaurants and mass catering, as Pardo and Jimenez [43] found in Spain and Christiansen et al. [42] in Brussels, with mislabeling rates up to 13% in restaurants and 25% of cod mislabeling in the mass catering sector in the whole of Europe [11].

A higher number of different substitute species can be detected when it comes to the inclusion of restaurants or takeaway shops in the studies [11,42,43,47]. Countries which have been sampled predominantly in supermarkets or fish markets do not show this trend [5]. In combination with the higher mislabeling rates in these selling points, it could imply active mislabeling, since EU labelling and traceability regulation are less strict for restaurants [26]. However, more studies should be conducted for confirmation.

Although there is an apparent reduction of mislabeling for the EU [5,30], certain substitute species remain frequently used through the years (Table 3). *Pollachius virens* and *M. aeglefinus,* as such, appears in seven years of a total of nine years analysed, including the current study. As one of the species with the highest substitution proportions re-occurring through the studies, *P. virens* is still listed as a species of least concern in the IUCN data base and with relatively low catching rates, with mean annual number of 349,026 tons in comparison to *G. morhua,* with mean annual catching number of 1,124,884 tons in the last decade. Therefore, the population of *P. virens* does not seem to be affected too much, but the available catch data is not accurate. Haddock (*M. aeglefinus*), on the other hand, also a frequently used substitute species in Denmark, Sweden, or France, as the only species found as substituted for cod, is listed as vulnerable in the IUCN database. Although its annual catch rate is similar to that of *P. virens*, this demonstrates how dangerous mislabeling can be for a specific population, since the catch is not reported and the stocks are already under pressure. The price of 2.79€/kilogram is also below the value of cod, which ranges between 4€ and 10.90€ (FAO February 2020). This could be a driving factor behind the mislabeling since less valuable fish can potentially be sold for a higher value. *G. chalcogrammus* also appears frequently as substitute species but proportionately less than *P. virens* and *M. aeglefinus*. This is despite the fact that the species was listed as near threatened in the IUCN database in 2013. However, its status changed in 2019 to not evaluated since it is often used as an ingredient in processed food, and its value has been reported as 1.51€ (FAO, February 2020); a current stock assessment is necessary. *G. macrocephalus* and *G. morhua* have also occurred as substitution species most frequently of each other. Some stores (Table 1), however, sell them together labelled as cod and stating both scientific names. These practices should be further regulated under the EU law since *G. macrocephalus* is not evaluated in the IUCN list, but *G. morhua* is listed as vulnerable. The official annual number of total catches for *G. macrocephalus* (391,292 tons) are also lower than for *G. morhua* (1,124,884 tons).

The cod market, as one of the most studied seafood species/products [21], shows positive signals towards successful increased control since the proportion of mislabeled cod is decreasing over the last decade despite the increase of studies. But not only should the detection of mislabeling be a responsibility of science, it should be implemented in the EU law to take routine controls in all levels; EU-wide normatives should be implemented to standardize those controls. Furthermore, new technologies like fisheries electronic monitor systems could be applied to help programmes based on inspectors and observers. Tools like facial recognition software could be adapted to recognize the fish species caught [48]. In addition, samples should be collected at every selling point regularly as standard control under EU regulation.

On the other hand, it is important to highlight the lack of information about the fishing methods used in samples obtained from fish markets for fresh cod products in Germany and the Netherlands since tags with more detailed information were missing and the fishmongers were often not informed either. This contests the standards of the Regulation (EU) No 1379/2013 by the CMO of the European Parliament and Council stating that processed or unprocessed food from fishery or aquaculture products need to be labelled or marked with the information on the exact species, how the species was produced, and its origin. Moreover, we found the use of fishery techniques declared as destructive by the European Union through the (CFP) Common Fisheries Policy [49], such as bottom otter trawling. Some products sampled for this study have their catch region labelled as FAO 27 or FAO 27 and FAO 67 when the product was labelled to consist either of *G. morhua* or *G. macrocephalus*. The area FAO 27 includes all the regions within the Northeast Atlantic, including catching areas of all European coastal countries from Portugal to Norway but also Iceland and Greenland [50]. The area FAO 67 consists of all marine waters bounded by a line commencing from a point at mainland Russia in the Eastern Bering Sea, crossing through Alaska and Canada and departing within USA territory [51]. However, not every country shares the same catch quotes [52]. Norway, Iceland, or Great Britain, for example, are not bound to EU regulation and thus have different fishing quotas. Cod stocks in the North Sea are still overexploited and therefore unsustainable [52], but this is not the case for the cod stocks of the Barents Sea [53]. Since both areas are covered by the FAO 27 area, it is not clear if the product comes from a sustainably managed stock or not. Sadly, the European Regulation EU 1379/2013, Art. 35 only specifies the area of fishing to be labelled and not the subarea. EU fishing vessels seeking to fish outside European waters must comply with the sustainable management of external fishing fleets regulation (SMEFF). However, the SMEFF stays silent when it comes to cooperation in the management of unregulated high sea stocks, includes poor monitoring practices (e.g., reflagging and chartering), lacks coherence regarding sustainability criteria, and does not provide public access to data on beneficial ownership [54]. This undermines the effort on traceability provided by the Regulation (EU) No 1379/2013 since any catch locations outside the EU (FAO 67 and certain areas within FAO 27 (e.g., Barents Sea)), falls under the SMEFF regulation.

Finally, only fifteen scientific investigations with complete, comparable information were found from 2008 to 2020 that checked labelling correctness of cod in the EU (Table 2). This, together with inconsistent sample sizes ranging from 6 to 546 samples and lack of data in 2009, 2011, 2018, and prior years, makes it difficult to determine a defined trend.

## 5. Conclusions

Over the last decade, the general trend of mislabeled cod within the EU is significantly negative, which is very positive for the conservation of *G. morhua* and *G. macrocephalus* and testifies of increased awareness, probably thanks to more publications and media coverage. This trend is positive, too, for their substitute species, especially for those listed as vulnerable, endangered, and critically endangered fish species. However, there are different trends in cod fillets in the three markets analysed, with a significant increment in French markets.

Therefore, although the general trend is decreasing, the differences found in different markets highlight the need of implementation of standard controls on different selling points and fish products.

## Figures and Tables

**Table 1:** Samples analyzed in this study and the information provided in the labels. Each sample was coded with the information of the fish, its condition (fresh/frozen), the country, city, and store of purchase. Fre, fresh; Fro, frozen; Ger, Germany; Net, the Netherlands; Fra, France; N/A, not available data. Supermarket/fish market names are not provided to keep confidentiality. (e.g., CodFroNetRA1 = Cod, Frozen, Netherlands, Rotterdam, store A).

Sample Number	Species in Label	Country	Number of Samples	City	Selling Point	Catch Method	Catch Location
CodFreGerHW	*G. morhua*	Germany	4	Hamburg	Fish market	trawl nets	Barents Sea (FAO 27)
CodFreGerHF	*G. morhua*	Germany	10	Hamburg	Fish market	trawl nets	Norwegian Sea (FAO 27)
CodFreGerBS	*G. morhua*	Germany	10	Bremerhaven	Fish market	trawl nets	Great Belt (FAO 27)
CodFreGerHAF	*G. morhua*	Germany	2	Hamburg	Fish market	N/A	N/A
CodFreGerFN	*G. morhua*	Germany	2	Fulda	Fish market chain	seine fishing	North Atlantic Ocean (FAO 27)
CodFreGerFE	*G. morhua*	Germany	3	Fulda	Supermarket	N/A	Norwegian Sea (FAO 27)
CodFreGerFT	*G. morhua*	Germany	4	Fulda	Supermarket	N/A	Iceland (FAO 27)
CodFroGerHL	*G. morhua*	Germany	6	Hamburg	Supermarket	various	FAO 27
CodFroGerHA	*G. morhua*	Germany	6	Hamburg	Supermarket	bottom otter trawl	Spitzbergen, Barents Sea (FAO 27)
CodFroGerFT	*G. morhua*	Germany	10	Fulda	Supermarket	various	Barents Sea/Norwegian Sea/North East Atlantic (FAO 27)
CodFroGerFR	*G. morhua*	Germany	8	Fulda	Supermarket	seine fishing/long lines	Barents Sea/Norwegian Sea (FAO 27)
CodFroGerFK	*G. morhua*	Germany	10	Fulda	Supermarket	trawl nets/NA	North East Atlantic/(FAO 27)
CodFroGerFL	*G. morhua/G. macrocephalus*	Germany	16	Fulda	Supermarket	various	North East Atlantic (FAO 27)/North East Pacific (FAO 67)
CodFroGerBA	*G. morhua*	Germany	2	Bremerhaven	Supermarket	bottom otter trawl	Barents Sea (FAO 27) Norwegian Sea
CodFroGerFA	*G. morhua*	Germany	8	Fulda	Supermarket	trawl nets	FAO 27
CodFreNetRVg	*G. morhua*	The Netherlands	10	Rotterdam	Fish market	N/A	North Sea (FAO 27)
CodFreNetBd	*G. morhua*	The Netherlands	14	Rotterdam	Fish market	N/A	Norway (FAO 27)
CodFreNetRN	*G. morhua*	The Netherlands	1	Rotterdam	Fish market	N/A	N/A
CodFreNetRVb	*G. morhua*	The Netherlands	5	Rotterdam	Fish market	N/A	North Sea (FAO 27)
CodFroNetRA	*G. morhua*	The Netherlands	21	Rotterdam	Supermarket	seine fishing/long lines	North East Atlantic, Barents Sea, North Sea (FAO 27)
CodFroNetRH	*G. morhua*	The Netherlands	20	Rotterdam	Supermarket	trawl nets, seine fishing/long lines	North East Atlantic, Barents Sea, North Sea (FAO 27)
CodFreFraMCf	*G. morhua*	France	3	Montpellier	Supermarket	hooks, long lines	Iceland, Faroe Islands (FAO 27)
CodFreFraMAu	*G. morhua*	France	5	Montpellier	Supermarket	trawls, hooks long lines	Faroe Islands, Barents Sea (FAO 27)
CodFreFraSCc	*G. morhua*	France	7	Sète	Fish market	trawls	FAO 27
CodFreFraSCl	*G. morhua*	France	8	Sète	Fish market	trawls	FAO 27
CodFreFraSPr	*G. morhua*	France	5	Sète	Fish market	trawls	FAO 27
CodFreFraSCk	*G. morhua*	France	3	Sète	Fish market	trawls	FAO 27
CodFroFraMCar	*G. morhua*	France	23	Montpellier	Supermarket	hooks, long lines, trawls	Barents Sea Faroe Islands FAO 27Iceland (FAO 27)
CodFroFraMSu	*G. morhua*	France	12	Montpellier	Supermarket	hooks, long lines, trawls	Barents Sea, Norwegian Sea, (FAO 27)
CodFroFraMAu	*G. morhua*	France	14	Montpellier	Supermarket	hooks, long lines, trawls	Barents Sea, Norwegian Sea, North Sea (FAO 27)

**Table 2:** Number of mislabeled cod within the EU from literature. Year of sampling, number of correctly and incorrectly labelled samples, country, species reported on the label, and the published reference are given.

Year of Sampling	Correctly Labelled	Incorrectly Labelled	Sampling Country	Species on Label	Reference
2008	4	2	Italy	*G. morhua*	Filonzi et al. 2010 (Appendix A) [37]
2010	94	37	Ireland	Cod	Miller and Mariani 2010 (Appendix A) [32]
2012	88	7	UK	Cod	Miller, Jessel, and Mariani 2012 (Appendix A) [28]
2013	43	62	Italy	Cod	Pinto et al. 2013 (Appendix A) [29]
2013	512	34	Spain	*G. morhua*	Helgoe et al. 2020 (Appendix A) [31]
2013	2	2	Italy	Cod	Cutarelli et al. 2013 (Appendix A) [38]
2013	136	6	France	Cod	Benard-Capelle et al. 2015 (Appendix A) [30]
2014	5	0	Portugal	*G. morhua/G. macrocephalus*	Harris et al. 2016 (Appendix A) [39]
2014	48	0	France	Cod	Mariani et al. 2015 (Appendix A) [5]
2014	42	0	Germany	Cod	Mariani et al. 2015 (Appendix A) [5]
2014	55	1	Ireland	Cod	Mariani et al. 2015 (Appendix A) [5]
2014	67	4	Portugal	Cod, *G. morhua/G. macrocephalus*	Mariani et al. 2015 (Appendix A) [5]
2014	118	4	Spain	Cod	Mariani et al. 2015 (Appendix A) [5]
2014	138	8	UK	Cod	Mariani et al. 2015 (Appendix A) [5]
2014	170	9	UK	Cod	Helyar et al. 2014 (Appendix A) [40]
2015	43	0	Norway	Cod	Brechon et al. 2016 (Appendix A) [25]
2015	44	0	Netherlands	Cod	Brechon et al. 2016 (Appendix A) [25]
2015	13	19	Estonia	Cod	Brechon et al. 2016 (Appendix A) [25]
2015	43	0	Belgium	Cod	Brechon et al. 2016 (Appendix A) [25]
2015	41	1	UK	Cod	Brechon et al. 2016 (Appendix A) [25]
2015	35	8	Denmark	Cod	Brechon et al. 2016 (Appendix A) [25]
2015	43	2	Sweden	Cod	Brechon et al. 2016 (Appendix A) [25]
2016	49	5	Spain	Cod	Muñoz-Colmenero et al. 2016 (Appendix A) [41]
2016	62	11	Belgium	Cod	Christiansen et al. 2018 (Appendix A) [42]
2016	41	6	Spain	*G. morhua*	Pardo and Jimenez 2020 (Appendix A) [43]
2017	7	3	Portugal	*G. Morhua/*Cod	Pardo et al. 2018 (Appendix A) [11]
2017	2	1	France	Cod	Pardo et al. 2018 (Appendix A) [11]
2017	1	1	Czech Republic	Cod	Pardo et al. 2018 (Appendix A) [11]
2017	1	1	Greece	Cod	Pardo et al. 2018 (Appendix A) [11]
2017	4	1	UK	*G. morhua/G. macrocephalus*	Pardo et al. 2018 (Appendix A) [11]
2017	0	1	Finland	*G. morhua/G. macrocephalus*	Pardo et al. 2018 (Appendix A) [11]
2017	1	1	Latvia	*G. morhua/G. macrocephalus*	Pardo et al. 2018 (Appendix A) [11]
2017	1	1	Switzerland	*G. morhua/G. macrocephalus*	Pardo et al. 2018 (Appendix A) [11]
2017	1	0	Germany	Cod	Pardo et al. 2018 (Appendix A) [11]
2017	2	0	Belgium	Cod	Pardo et al. 2018 (Appendix A) [11]
2017	3	0	Norway	Cod	Pardo et al. 2018 (Appendix A) [11]
2017	2	0	Italy	Cod	Pardo et al. 2018 (Appendix A) [11]
2017	1	0	Netherlands	Cod	Pardo et al. 2018 (Appendix A) [11]
2017	3	0	Ireland	*G. morhua*	Pardo et al. 2018 (Appendix A) [11]
2018	10	0	Greece	Cod	Minoudi et al. 2020 (Appendix A) [44]
2019	70	4	UK, Belgium	*G. macrocephalus/G.morhua*	Barendse et al. 2019 (Appendix A) [45]
2019	42	1	UK	Cod	Barendse et al. 2019 (Appendix A) [45]
2019	111	3	Belgium	*G. morhua*	Deconinck et al. 2020 (Appendix A) [24]
2020	71	0	Netherlands	*G. morhua*	This study
2020	101	0	Germany	*G. morhua/G. macrocephalus*	This study
2020	27	9	France	*G. morhua*	This study

**Table 3:** The years each substitute species was found (*G. morhua* was substitute species of *G. macrocephalus*). IUCN, International Union for Conservation of Nature.

Substitute Species	IUCN Status	Years of Occurrence
*Gadus morhua*	Vulnerable	2010, 2012, 2014, 2019
*G. macrocephalus*	Not evaluated	2013, 2014, 2019
*Lates niloticus*	Least concern	2013, 2016
*G. chalcogrammus*	Near threatened	2013, 2015, 2016, 2017, 2019
*Molva molva*	Data deficient	2013, 2014, 2016, 2017
*Pangasianodon hypophtalmus*	Endangered	2012, 2013, 2017
*Pollachius pollachius*	Least concern	2010, 2016
*P. virens*	Least concern	2010, 2013, 2015, 2016, 2017, 2019
*Melanogrammus aeglefinus*	Vulnerable	2010, 2012, 2013, 2014, 2015, 2019, 2020

## Data Availability

Accession numbers of all analyzed sequences are present into the text.

## Appendix A

**Table A1:** Accession Numbers from the Publications Used in the Study, If Provided.

Publication	Sequence Accession Number
Filonzi et al., 2010 [38]	GenBank: EU7521, DQ173995, EU752090, EF211102
Miller and Mariani, 2010 [32]	not provided
Miller, Jessel, and Mariani, 2012 [28]	not provided
Pinto et al., 2013 [29]	not provided
Helgoe et al., 2020 [30]	not provided
Cutarelli et al., 2013 [39]	not provided
Benard-Capelle et al. 2015 [33]	Barcode of Life: FCSF001-14 to FCSF291-14; FCSF292-14 to FCSF404-14
Harris et al. 2016 [40]	not provided
Mariani et al., 2015 [32]	GenBank: KJ510424–KJ531384; KJ563141–KJ645864
Helyar et al., 2014 [41]	GenBank: KJ614671--KJ615069
Brechon et al., 2016 [25]	not provided
Muñoz-Colmenero et al., 2016 [42]	GenBank: KF597025–KF597028; KF597030; KF597040–KF597046; KF597048; KP330299–KP330373; KP330374–KP330387
Christiansen et al., 2018 [43]	not provided
Pardo and Jimenez, 2020 [44]	GenBank: MT266987-MT267272
Pardo et al., 2018 [11]	not provided
Minoudi et al., 2020 [45]	not provided
Barendse et al., 2019 [46]	not provided
Deconinck et al., 2020 [24]	not provided

## References

[1] Galal-Khallaf A., Ardura A., Mohammed-Geba K., Borrell Y.J., Garcia-Vazquez E. (2014). DNA Barcoding Reveals a High Level of Mislabelling in Egyptian Fish Fillets. Food Control.

[2] Khaksar R., Carlson T., Schaffner D.W., Ghorashi M., Best D., Jandhyala S., Traverso J., Amini S. (2015). Unmasking Seafood Mislabelling in U.S. Markets: DNA Barcoding as a Unique Technology for Food Authentication and Quality Control. Food Control.

[3] Carvalho D.C., Guedes D., da Gloria Trindade M., Coelho R.M.S., de Lima Araujo P.H. (2017). Nationwide Brazilian Governmental Forensic Programme Reveals Seafood Mislabelling Trends and Rates Using DNA Barcoding. Fish. Res..

[4] Xiong X., Guardone L., Cornax M.J., Tinacci L., Guidi A., Gianfaldoni D., Armani A. (2016). DNA barcoding reveals substitution of sablefish (Anoplopoma fimbria) with Patagonian and Antarctic toothfish (*Dissostichus eleginoides* and *Dissostichus mawsoni*) in online market in China: How mislabeling opens door to IUU fishing. Food Control.

[5] Mariani S., Griffiths A.M., Velasco A., Kappel K., Jerome M., Perez-Martin R.I., Schroder U., Verrez-Bagnis V., Silva H., Vandamme S.G. (2015). Low Mislabelling Rates Indicate Marked Improvements in European Seafood Market Operations. Front. Ecol. Environ..

[6] PDF4PRO. https://pdf4pro.com/view/the-state-of-world-fisheries-and-aquaculture-187a.html.

[7] European Commission https://ec.europa.eu/fisheries/press/eu-fish-market-2019-edition-out-everything-you-wanted-know-about-eu-market-fish-and-seafood_en.

[8] EUMOFA https://www.eumofa.eu/market-analysis#yearly.

[9] European Commission https://ec.europa.eu/fisheries/eumofa-monthly-highlights-12018_en.

[10] Olsgard F., Schaanning M.T., Widdicombe S., Kendall M.A., Austen M.C. (2008). Effects of Bottom Trawling on Ecosystem Functioning. J. Exp. Mar. Biol. Ecol..

[11] Pardo M.Á., Jiménez E., Viðarsson J.R., Ólafsson K., Ólafsdóttir G., Daníelsdóttir A.K., Pérez-Villareal B. (2018). DNA Barcoding Revealing Mislabelling of Seafood in European Mass Caterings. Food Control.

[12] Pardo M.Á., Jiménez E., Pérez-Villarreal B. (2016). Misdescription Incidents in Seafood Sector. Food Control.

[13] Kroetz K., Luque G.M., Gephart J.A., Jardine S.L., Lee P., Moore K.C., Cole C., Steinkruger A., Josh Donlan C. (2020). Consequences of Seafood Mislabelling for Marine Populations and Fisheries Management. Proc. Natl. Acad. Sci. USA.

[14] Triantafyllidis A., Karaiskou N., Perez J., Martinez J.L., Roca A., Lopez B., Garcia-Vazquez E. (2010). Fish Allergy Risk Derived from Ambiguous Vernacular Fish Names: Forensic DNA-Based Detection in Greek Markets. Food Res. Int..

[15] Barcaccia G., Lucchin M., Cassandro M. (2016). DNA Barcoding as a Molecular Tool to Track down Mislabelling and Food Piracy. Diversity.

[16] Kochzius M., Seidel C., Antoniou A., Botla S.K., Campo D., Cariani A., Vazquez E.G., Hauschild J., Hervet C., Hjörleifsdottir S. (2010). Identifying Fishes through DNA Barcodes and Microarrays. PLoS ONE.

[17] Huxley-Jones E., Shaw J.L.A., Fletcher C., Parnell J., Watts P.C. (2012). Use of DNA Barcoding to Reveal Species Composition of Convenience Seafood. Conserv. Biol..

[18] Hebert P.D.N., Cywinska A., Ball S.L., de Waard J.R. (2003). Biological identifications through DNA barcodes. Proc. Natl. Acad. Sci. USA.

[19] Ward R., Hanner R., Hebert P. (2009). The campaign to DNA barcode all fishes, FISH-BOL. J. Fish Biol..

[20] Hebert P.D.N., Ratnasingham S., DeWaard J.R. (2003). Barcoding Animal Life: Cytochrome c Oxidase Subunit 1 Divergences among Closely Related Species. Proc. R. Soc. B Biol. Sci..

[21] Naaum A.M., Warner K., Mariani S., Hanner R.H., Carolin C.D. (2016). Seafood Mislabelling Incidence and Impacts.

[22] Standal D., Bouwer Utne I. (2007). Can Cod Farming Affect Cod Fishing? A System Evaluation of Sustainability. Mar. Policy.

[23] Halldórsson J.E., Björnsson B., Gunnlaugsson S.B. (2012). Feasibility of Ranching Coastal Cod (*Gadus morhua*) Compared with on-Growing, Full-Cycle Farming and Fishing. Mar. Policy.

[24] Deconinck D., Volckaert F.A.M., Hostens K., Panicz R., Eljasik P., Faria M., Monteiro C.S., Robbens J., Derycke S. (2020). A High-Quality Genetic Reference Database for European Commercial Fishes Reveals Substitution Fraud of Processed Atlantic Cod (*Gadus morhua*) and Common Sole (*Solea solea*) at Different Steps in the Belgian Supply Chain. Food Chem. Toxicol..

[25] Bréchon A.L., Hanner R., Mariani S. (2016). A Systematic Analysis across North Atlantic Countries Unveils Subtleties in Cod Product Labelling. Mar. Policy.

[26] Kappel K., Schröder U. (2016). Substitution of High-Priced Fish with Low-Priced Species: Adulteration of Common Sole in German Restaurants. Food Control.

[27] Günther B., Raupach M.J., Knebelsberger T. (2017). Full-length and mini-length DNA barcoding for the identification of seafood commercially traded in Germany. Food Control.

[28] Miller D., Jessel A., Mariani S. (2012). Seafood Mislabelling: Comparisons of Two Western European Case Studies Assist in Defining Influencing Factors, Mechanisms and Motives. Fish Fish..

[29] Di Pinto A., Di Pinto P., Terio V., Bozzo G., Bonerba E., Ceci E., Tantillo G. (2013). DNA Barcoding for Detecting Market Substitution in Salted Cod Fillets and Battered Cod Chunks. Food Chem..

[30] Bénard-Capelle J., Guillonneau V., Nouvian C., Fournier N., Le Loët K., Dettai A. (2015). Fish mislabelling in France: Substitution rates and retail types. PeerJ.

[31] Helgoe J., Oswald K.J., Quattro J.M. (2020). A Comprehensive Analysis of the Mislabelling of Atlantic Cod (*Gadus morhua*) Products in Spain. Fish. Res..

[32] Miller D.D., Mariani S. (2010). Smoke, Mirrors, and Mislabelled Cod: Poor Transparency in the European Seafood Industry. Front. Ecol. Environ..

[33] Estoup A., Largiader C.R., Perrot E., Chourrout D. (1996). Rapid one-tube DNA extraction for reliable PCR dectection of fish polymorphic markers and transgenes. Mol. Mar. Biol. Biotechnol..

[34] Handy S.M., Deeds J.R., Ivanova N.V., Hebert P.D., Hanner R.H., Ormos A., Yancy H.F. (2011). A single-laboratory validated method for the generation of DNA barcodes for the identification of fish for regulatory compliance. J. AOAC Int..

[35] Ward R.D., Zemlak T.S., Innes B.H., Last P.R., Hebert P.D. (2005). DNA barcoding Australia’s fish species. Philos. Trans. R. Soc. B Biol. Sci..

[36] Hall T.A. (1999). BioEdit: A user-friendly biological sequence alignment editor and analysis program for Windows 95/98/NT. Nucleic Acids Symposium Series.

[37] Filonzi L., Chiesa S., Vaghi M., Nonnis Marzano F. (2010). Molecular Barcoding Reveals Mislabelling of Commercial Fish Products in Italy. Food Res. Int..

[38] Cutarelli A., Amoroso M.G., De Roma A., Girardi S., Galiero G., Guarino A., Corrado F. (2014). Italian Market Fish Species Identification and Commercial Frauds Revealing by DNA Sequencing. Food Control.

[39] Harris D.J., Rosado D., Xavier R. (2016). DNA Barcoding Reveals Extensive Mislabelling in Seafood Sold in Portuguese Supermarkets. J. Aquat. Food Prod. Technol..

[40] Helyar S.J., Lloyd H.D., De Bruyn M., Leake J., Bennett N., Carvalho G.R. (2014). Fish Product Mislabelling: Failings of Traceability in the Production Chain and Implications for Illegal, Unreported and Unregulated (IUU) Fishing. PLoS ONE.

[41] Muñoz-Colmenero M., Blanco O., Arias V., Martinez J.L., Garcia-Vazquez E. (2016). L’authentification ADN Des Produits Halieutiques Révèle Un Mauvais Étiquetage Associé Au Traitement Des Fruits de Mer. Fisheries.

[42] Christiansen H., Fournier N., Hellemans B., Volckaert F.A.M. (2018). Seafood Substitution and Mislabelling in Brussels’ Restaurants and Canteens. Food Control.

[43] Pardo M.Á., Jiménez E. (2020). DNA Barcoding Revealing Seafood Mislabelling in Food Services from Spain. J. Food Compos. Anal..

[44] Minoudi S., Karaiskou N., Avgeris M., Gkagkavouzis K., Tarantili P., Triantafyllidou D., Palilis L., Avramopoulou V., Tsikliras A., Barmperis K. (2020). Seafood Mislabelling in Greek Market Using DNA Barcoding. Food Control.

[45] Barendse J., Roel A., Longo C., Andriessen L., Webster L.M.I., Ogden R., Neat F. (2019). DNA Barcoding Validates Species Labelling of Certified Seafood. Curr. Biol..

[46] Mariani S., Ellis J., O’Reilly A., Bréchon A.L., Sacchi C., Miller D.D. (2014). Mass Media Influence and the Regulation of Illegal Practices in the Seafood Market. Conserv. Lett..

[47] Horreo J.L., Fitze P.S., Jiménez-Valverde A., Noriega J.A., Pelaez M.L. (2019). Amplification of 16S RDNA Reveals Important Fish Mislabelling in Madrid Restaurants. Food Control.

[48] Gilman E., Legorburu G., Fedoruk A., Heberer C., Zimring M., Barkai A. (2019). Increasing the Functionalities and Accuracy of Fisheries Electronic Monitoring Systems. Aquat. Conserv. Mar. Freshw. Ecosyst..

[49] Vlachopoulou E.I., Wilson A.M., Miliou A. (2013). Disconnects in EU and Greek Fishery Policies and Practices in the Eastern Aegean Sea and Impacts on Posidonia Oceanica Meadows. Ocean Coast. Manag..

[50] Food and Agriculture Organization of the United Nations http://www.fao.org/fishery/area/Area27/en.

[51] Food and Agriculture Organization of the United Nations http://www.fao.org/tempref/FI/maps/fig_h4_67_0.gif.

[52] Pedersen E.J., Thompson P.L., Ball R.A., Fortin M.J., Gouhier T.C., Link H., Moritz C., Nenzen H., Stanley R.R.E., Taranu Z.E. (2017). Signatures of the Collapse and Incipient Recovery of an Overexploited Marine Ecosystem. R. Soc. Open Sci..

[53] Årthun M., Bogstad B., Daewel U., Keenlyside N.S., Sandø A.B., Schrum C., Ottersen G. (2018). Climate Based Multi-Year Predictions of the Barents Sea Cod Stock. PLoS ONE.

[54] Guggisberg S. (2019). The EU’s Regulation on the Sustainable Management of External Fishing Fleets: International and European Law Perspectives. Int. J. Mar. Coast. Law.

